# 2-{(*E*)-[(2*Z*)-(3-Chloro-1-methyl-2,2-di­oxo-3,4-dihydro-1*H*-2,1-benzo­thia­zin-4-yl­idene)hydrazinyl­idene]meth­yl}phenol

**DOI:** 10.1107/S1600536811055978

**Published:** 2012-01-07

**Authors:** Muhammad Shafiq, Islam Ullah Khan, Muhammad Zia-ur-Rehman, Muhammad Nadeem Arshad, Abdullah M. Asiri

**Affiliations:** a Materials Chemistry Laboratory, Department of Chemistry, GC University, Lahore 54000, Pakistan; b Applied Chemistry Research Center, PCSIR Laboratories Complex, Ferozpur Road, Lahore 54600, Pakistan; c Department of Chemistry, University of Gujrat, Gujrat 50781, Pakistan; d The Center of Excellence for Advanced Materials Research and Department of Chemistry, Faculty of Science, King Abdul Aziz University, Jeddah, PO Box 80203, Saudi Arabia

## Abstract

In the title compound, C_16_H_14_ClN_3_O_3_S, the thia­zine ring adopts a sofa (half-chair) conformation, with an r.m.s. deviation from the mean plane of 0.23 Å. The S atom and S-bonded C atom exhibit the maximum deviations from the thia­zine mean plane [−0.3976 (12) and 0.3179 (14) Å, respectively]. The conformations around the double bonds in the *R*_2_C=N—N=CH*R* unit are *Z* and *E*. An intra­molecular O—H⋯N hydrogen bond with the hy­droxy group as donor generates an *S*(6) ring motif. In the crystal, pairs of weak C—H⋯O inter­actions connect the mol­ecules, forming inversion dimers.

## Related literature

For benzothia­zine compounds, see: Shafiq, Khan *et al.* (2011 ▶); Shafiq, Zia-ur-Rehman *et al.* (2011 ▶). For related structures, see: Shafiq *et al.* (2011*a* ▶,*b* ▶). For graph-set notation, see: Bernstein *et al.* (1995 ▶).
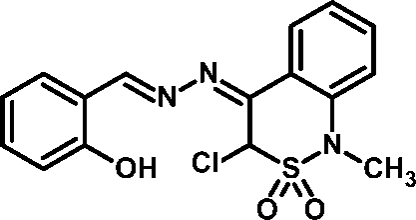


## Experimental

### 

#### Crystal data

C_16_H_14_ClN_3_O_3_S
*M* *_r_* = 363.81
Monoclinic, 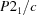

*a* = 7.0973 (5) Å
*b* = 12.0957 (7) Å
*c* = 18.7396 (13) Å
β = 96.058 (4)°
*V* = 1599.75 (18) Å^3^
*Z* = 4
Mo *K*α radiation
μ = 0.39 mm^−1^
*T* = 296 K
0.19 × 0.08 × 0.07 mm

#### Data collection

Bruker Kappa APEXII CCD diffractometer
Absorption correction: multi-scan (*SADABS*; Bruker, 2007 ▶) *T*_min_ = 0.930, *T*_max_ = 0.973
15526 measured reflections
3977 independent reflections
2200 reflections with *I* > 2σ(*I*)
*R*_int_ = 0.061

#### Refinement

*R*[*F* ^2^ > 2σ(*F* ^2^)] = 0.073
*wR*(*F* ^2^) = 0.211
*S* = 1.03
3977 reflections
221 parameters
H atoms treated by a mixture of independent and constrained refinement
Δρ_max_ = 1.13 e Å^−3^
Δρ_min_ = −0.38 e Å^−3^

### 

Data collection: *APEX2* (Bruker, 2007 ▶); cell refinement: *SAINT* (Bruker, 2007 ▶); data reduction: *SAINT*; program(s) used to solve structure: *SHELXS97* (Sheldrick, 2008 ▶); program(s) used to refine structure: *SHELXL97* (Sheldrick, 2008 ▶); molecular graphics: *ORTEP-3 for Windows* (Farrugia, 1997 ▶) and *PLATON* (Spek, 2009 ▶); software used to prepare material for publication: *WinGX* (Farrugia, 1999 ▶) and *PLATON*.

## Supplementary Material

Crystal structure: contains datablock(s) I, global. DOI: 10.1107/S1600536811055978/bh2407sup1.cif

Structure factors: contains datablock(s) I. DOI: 10.1107/S1600536811055978/bh2407Isup2.hkl

Supplementary material file. DOI: 10.1107/S1600536811055978/bh2407Isup3.cml

Additional supplementary materials:  crystallographic information; 3D view; checkCIF report

## Figures and Tables

**Table 1:** Hydrogen-bond geometry (Å, °)

*D*—H⋯*A*	*D*—H	H⋯*A*	*D*⋯*A*	*D*—H⋯*A*
O3—H3*O*⋯N3	0.82 (7)	1.98 (7)	2.682 (5)	143 (7)
C9—H9⋯O1^i^	0.95	2.55	3.394 (5)	148

Symmetry code: (i) 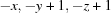.

**Fig. 1.**
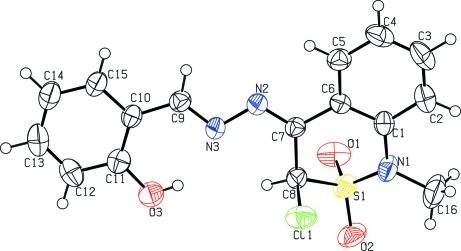
The molecular structure of the title compound showing 50% displacement ellipsoids.

**Fig. 2.**
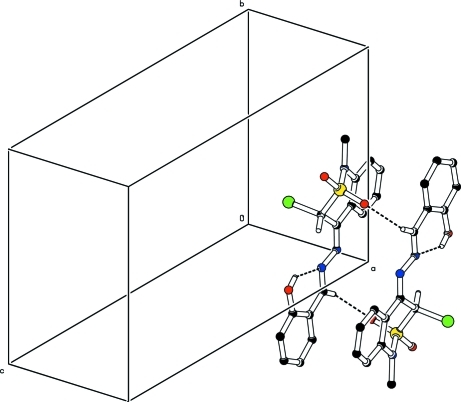
Perspective view which shows the dimers formed through C—H···O hydrogen bonds (dashed lines).

## supplementary crystallographic information

### Comment

We have recently explored the synthesis of different halogenated benzothiazines (Shafiq, Khan, Arshad *et al.*, 2011), and their Schiff bases (Shafiq, Zia-ur-Rehman *et al.*, 2011). The crystal structure of title compound is being reported in order to study the geometry and different interactions in this class of compounds.

The present structure relates with the already published crystal structures of 4-hydrazinylidene-1-methyl-3*H*-2λ^6^,1-benzothiazine-2,2-dione (Shafiq, Khan, Zia-ur-Rehman *et al.*, 2011*a*) and 6-bromo-1-methyl-4-[2-(4-methylbenzylidene)hydrazinylidene]-3*H*-2λ^6^,1-benzothiazine-2,2-dione (Shafiq, Khan, Zia-ur-Rehman *et al.*, 2011*b*). The two fused rings in the title compound (Fig. 1) are oriented at dihedral angle of 7.49 (5)° and the thiazine ring adopts the sofa shape with r.m.s. deviation of about 0.23 Å, and with the maximum deviations arising from S1 [-0.3721 (21) Å] and C8 [0.3118 (26) Å] atoms. The intramolecular hydrogen bonding interaction of O—H···N type generates a six membered ring *S*_1_^1^(6) (Bernstein *et al.*, 1995). A weak C—H···O type interaction connects the molecules to form centrosymmetric dimers and generates *R*_2_^2^(16) ring motifs (Bernstein *et al.*, 1995; Table 1 and Fig. 2).

The phenol ring is oriented at dihedral angle of 8.17 (4) and 15.58 (5)° with respect to the aromatic ring and thiazine ring, and is twisted by 2.07 (3)° with respect to six membered *S*(6) ring motif generated through the intramolecular O—H···N hydrogen bond.

### Experimental

For the synthesis of title compound, 4-hydrazinylidene-1- methyl-3*H*-2λ^6^,1-benzothiazine-2,2-dione (Shafiq, Khan, Zia-ur-Rehman *et al.*, 2011*a*) was subjected to react with salicylaldehyde according to literature procedure (Shafiq, Zia-ur-Rehman *et al.*, 2011). The product obtained was then halogenated following another method (Shafiq, Khan, Arshad *et al.*, 2011). Suitable crystals were produced by slow evaporation of a dry ethylacetate solution.

### Refinement

All C-bonded H-atoms were positioned in idealized geometry, with C—H = 0.95 Å for aromatic CH, C—H = 0.98 Å for the methyl group, and C—H = 1 Å for methine C8, and were refined using a riding model with *U*_iso_(H) = 1.2*U*_eq_(C) for aromatic groups and C8, and *U*_iso_(H) = 1.5*U*_eq_(C16) for the methyl group. Hydroxyl H atom H3O was found in a difference map and refined freely, restraining the O—H bond length to 0.82 (7) Å, with *U*_iso_(H3O) = 2*U*_eq_(O3).

### Crystal data

**Table:**

C_16_H_14_ClN_3_O_3_S	*F*(000) = 752
*M**_r_* = 363.81	*D*_x_ = 1.511 Mg m^−^^3^
Monoclinic, *P*2_1_/*c*	Mo *K*α radiation, λ = 0.71073 Å
Hall symbol: -P 2ybc	Cell parameters from 2110 reflections
*a* = 7.0973 (5) Å	θ = 2.8–23.7°
*b* = 12.0957 (7) Å	µ = 0.39 mm^−^^1^
*c* = 18.7396 (13) Å	*T* = 296 K
β = 96.058 (4)°	Needle, colourless
*V* = 1599.75 (18) Å^3^	0.19 × 0.08 × 0.07 mm
*Z* = 4	

### Data collection

**Table:**

Bruker Kappa APEXII CCD diffractometer	3977 independent reflections
Radiation source: fine-focus sealed tube	2200 reflections with *I* > 2σ(*I*)
graphite	*R*_int_ = 0.061
φ and ω scans	θ_max_ = 28.3°, θ_min_ = 2.9°
Absorption correction: multi-scan (*SADABS*; Bruker, 2007)	*h* = −8→9
*T*_min_ = 0.930, *T*_max_ = 0.973	*k* = −16→16
15526 measured reflections	*l* = −24→25

### Refinement

**Table:**

Refinement on *F*^2^	Primary atom site location: structure-invariant direct methods
Least-squares matrix: full	Secondary atom site location: difference Fourier map
*R*[*F*^2^ > 2σ(*F*^2^)] = 0.073	Hydrogen site location: inferred from neighbouring sites
*wR*(*F*^2^) = 0.211	H atoms treated by a mixture of independent and constrained refinement
*S* = 1.03	*w* = 1/[σ^2^(*F*_o_^2^) + (0.0873*P*)^2^ + 1.6824*P*] where *P* = (*F*_o_^2^ + 2*F*_c_^2^)/3
3977 reflections	(Δ/σ)_max_ < 0.001
221 parameters	Δρ_max_ = 1.13 e Å^−^^3^
0 restraints	Δρ_min_ = −0.38 e Å^−^^3^
0 constraints	

### Fractional atomic coordinates and isotropic or equivalent isotropic displacement parameters (Å^2^)

**Table:**

	*x*	*y*	*z*	*U*_iso_*/*U*_eq_	
Cl1	0.51356 (17)	0.73641 (10)	0.42510 (7)	0.0613 (4)	
S1	0.16021 (16)	0.83188 (8)	0.46237 (6)	0.0466 (3)	
O1	−0.0316 (4)	0.7950 (3)	0.46762 (18)	0.0589 (9)	
O2	0.1931 (5)	0.9186 (2)	0.41328 (19)	0.0646 (10)	
O3	0.2121 (6)	0.4788 (3)	0.27384 (18)	0.0632 (10)	
N1	0.2779 (5)	0.8598 (3)	0.5392 (2)	0.0486 (9)	
N2	0.2405 (5)	0.5211 (3)	0.48831 (18)	0.0410 (8)	
N3	0.2244 (5)	0.4863 (2)	0.41731 (18)	0.0409 (8)	
C1	0.2935 (6)	0.7761 (3)	0.5927 (2)	0.0393 (9)	
C2	0.3168 (7)	0.8064 (4)	0.6654 (2)	0.0546 (12)	
H2	0.3185	0.8823	0.6784	0.066*	
C3	0.3368 (7)	0.7278 (5)	0.7172 (3)	0.0625 (14)	
H3	0.3541	0.7496	0.7662	0.075*	
C4	0.3327 (7)	0.6172 (4)	0.7003 (2)	0.0576 (12)	
H4	0.3462	0.5629	0.7371	0.069*	
C5	0.3090 (6)	0.5861 (4)	0.6295 (2)	0.0466 (10)	
H5	0.3059	0.5097	0.6178	0.056*	
C6	0.2894 (5)	0.6638 (3)	0.5745 (2)	0.0349 (8)	
C7	0.2647 (5)	0.6248 (3)	0.4995 (2)	0.0340 (8)	
C8	0.2740 (6)	0.7083 (3)	0.4402 (2)	0.0402 (9)	
H8	0.2059	0.6776	0.3950	0.048*	
C9	0.2063 (6)	0.3806 (3)	0.4143 (2)	0.0399 (9)	
H9	0.2041	0.3408	0.4579	0.048*	
C10	0.1889 (5)	0.3193 (3)	0.3476 (2)	0.0383 (9)	
C11	0.1917 (6)	0.3688 (3)	0.2807 (2)	0.0448 (10)	
C12	0.1740 (7)	0.3036 (4)	0.2194 (3)	0.0570 (12)	
H12	0.1764	0.3368	0.1736	0.068*	
C13	0.1532 (7)	0.1921 (4)	0.2249 (3)	0.0628 (14)	
H13	0.1388	0.1487	0.1824	0.075*	
C14	0.1526 (7)	0.1407 (4)	0.2903 (3)	0.0568 (12)	
H14	0.1393	0.0628	0.2932	0.068*	
C15	0.1715 (6)	0.2043 (3)	0.3513 (3)	0.0464 (10)	
H15	0.1728	0.1695	0.3968	0.056*	
C16	0.3125 (10)	0.9768 (4)	0.5597 (3)	0.0839 (18)	
H16A	0.2108	1.0030	0.5869	0.126*	
H16B	0.3153	1.0217	0.5163	0.126*	
H16C	0.4343	0.9831	0.5893	0.126*	
H3O	0.235 (11)	0.509 (6)	0.313 (4)	0.126*	

### Atomic displacement parameters (Å^2^)

**Table:**

	*U*^11^	*U*^22^	*U*^33^	*U*^12^	*U*^13^	*U*^23^
Cl1	0.0542 (7)	0.0619 (7)	0.0709 (9)	0.0012 (6)	0.0213 (6)	0.0147 (6)
S1	0.0503 (7)	0.0366 (5)	0.0514 (7)	0.0043 (5)	−0.0005 (5)	0.0050 (5)
O1	0.0359 (17)	0.073 (2)	0.066 (2)	0.0084 (15)	−0.0010 (14)	0.0259 (17)
O2	0.086 (3)	0.0398 (17)	0.066 (2)	−0.0017 (16)	−0.0010 (18)	0.0182 (15)
O3	0.094 (3)	0.0417 (18)	0.054 (2)	0.0080 (17)	0.0063 (19)	0.0097 (15)
N1	0.060 (2)	0.0312 (17)	0.052 (2)	0.0033 (16)	−0.0057 (17)	−0.0064 (15)
N2	0.049 (2)	0.0353 (17)	0.038 (2)	−0.0009 (15)	0.0044 (15)	−0.0005 (14)
N3	0.052 (2)	0.0315 (16)	0.040 (2)	−0.0002 (14)	0.0060 (15)	−0.0033 (14)
C1	0.035 (2)	0.041 (2)	0.041 (2)	0.0017 (17)	−0.0009 (17)	−0.0064 (18)
C2	0.055 (3)	0.062 (3)	0.045 (3)	0.001 (2)	0.001 (2)	−0.016 (2)
C3	0.058 (3)	0.095 (4)	0.034 (3)	−0.005 (3)	0.004 (2)	−0.015 (3)
C4	0.061 (3)	0.075 (3)	0.037 (3)	−0.004 (2)	0.004 (2)	0.011 (2)
C5	0.051 (3)	0.046 (2)	0.042 (3)	−0.0043 (19)	0.0007 (19)	0.0059 (19)
C6	0.032 (2)	0.040 (2)	0.033 (2)	−0.0003 (16)	0.0024 (15)	0.0014 (17)
C7	0.036 (2)	0.0306 (18)	0.035 (2)	0.0025 (15)	0.0018 (16)	0.0031 (16)
C8	0.051 (2)	0.0322 (19)	0.037 (2)	0.0031 (17)	0.0035 (18)	0.0001 (17)
C9	0.042 (2)	0.035 (2)	0.043 (2)	0.0007 (17)	0.0047 (18)	0.0022 (17)
C10	0.037 (2)	0.0342 (19)	0.043 (2)	0.0021 (16)	0.0013 (17)	−0.0020 (17)
C11	0.042 (2)	0.042 (2)	0.050 (3)	0.0069 (18)	−0.0001 (19)	0.0010 (19)
C12	0.061 (3)	0.069 (3)	0.040 (3)	0.007 (2)	0.000 (2)	−0.006 (2)
C13	0.059 (3)	0.063 (3)	0.066 (3)	0.003 (2)	0.004 (2)	−0.026 (3)
C14	0.056 (3)	0.043 (2)	0.072 (3)	−0.005 (2)	0.011 (2)	−0.015 (2)
C15	0.046 (3)	0.035 (2)	0.059 (3)	−0.0032 (18)	0.007 (2)	−0.007 (2)
C16	0.123 (5)	0.040 (3)	0.086 (4)	−0.004 (3)	−0.005 (4)	−0.016 (3)

### Geometric parameters (Å, °)

**Table:**

Cl1—C8	1.786 (4)	C5—C6	1.391 (5)
S1—O2	1.430 (3)	C5—H5	0.9500
S1—O1	1.445 (3)	C6—C7	1.475 (5)
S1—N1	1.623 (4)	C7—C8	1.509 (5)
S1—C8	1.770 (4)	C8—H8	1.0000
O3—C11	1.346 (5)	C9—C10	1.447 (5)
O3—H3O	0.82 (7)	C9—H9	0.9500
N1—C1	1.420 (5)	C10—C11	1.391 (6)
N1—C16	1.479 (6)	C10—C15	1.399 (5)
N2—C7	1.280 (5)	C11—C12	1.388 (6)
N2—N3	1.389 (5)	C12—C13	1.362 (7)
N3—C9	1.286 (5)	C12—H12	0.9500
C1—C6	1.399 (5)	C13—C14	1.374 (7)
C1—C2	1.405 (6)	C13—H13	0.9500
C2—C3	1.355 (7)	C14—C15	1.372 (6)
C2—H2	0.9500	C14—H14	0.9500
C3—C4	1.375 (7)	C15—H15	0.9500
C3—H3	0.9500	C16—H16A	0.9800
C4—C5	1.371 (6)	C16—H16B	0.9800
C4—H4	0.9500	C16—H16C	0.9800
			
O2—S1—O1	119.2 (2)	C7—C8—S1	109.6 (3)
O2—S1—N1	108.3 (2)	C7—C8—Cl1	111.1 (3)
O1—S1—N1	113.8 (2)	S1—C8—Cl1	110.0 (2)
O2—S1—C8	111.0 (2)	C7—C8—H8	108.7
O1—S1—C8	102.2 (2)	S1—C8—H8	108.7
N1—S1—C8	100.35 (19)	Cl1—C8—H8	108.7
C11—O3—H3O	111 (5)	N3—C9—C10	123.1 (4)
C1—N1—C16	120.1 (4)	N3—C9—H9	118.4
C1—N1—S1	118.1 (3)	C10—C9—H9	118.4
C16—N1—S1	119.0 (3)	C11—C10—C15	118.8 (4)
C7—N2—N3	116.8 (3)	C11—C10—C9	123.3 (4)
C9—N3—N2	110.0 (3)	C15—C10—C9	117.9 (4)
C6—C1—C2	119.1 (4)	O3—C11—C12	118.9 (4)
C6—C1—N1	121.5 (3)	O3—C11—C10	121.6 (4)
C2—C1—N1	119.4 (4)	C12—C11—C10	119.5 (4)
C3—C2—C1	120.3 (4)	C13—C12—C11	120.1 (5)
C3—C2—H2	119.8	C13—C12—H12	119.9
C1—C2—H2	119.8	C11—C12—H12	119.9
C2—C3—C4	121.3 (4)	C12—C13—C14	121.7 (5)
C2—C3—H3	119.4	C12—C13—H13	119.2
C4—C3—H3	119.4	C14—C13—H13	119.2
C5—C4—C3	119.1 (4)	C15—C14—C13	118.7 (4)
C5—C4—H4	120.4	C15—C14—H14	120.6
C3—C4—H4	120.4	C13—C14—H14	120.6
C4—C5—C6	121.6 (4)	C14—C15—C10	121.2 (4)
C4—C5—H5	119.2	C14—C15—H15	119.4
C6—C5—H5	119.2	C10—C15—H15	119.4
C5—C6—C1	118.5 (4)	N1—C16—H16A	109.5
C5—C6—C7	118.8 (3)	N1—C16—H16B	109.5
C1—C6—C7	122.7 (3)	H16A—C16—H16B	109.5
N2—C7—C6	118.1 (3)	N1—C16—H16C	109.5
N2—C7—C8	123.4 (3)	H16A—C16—H16C	109.5
C6—C7—C8	118.5 (3)	H16B—C16—H16C	109.5
			
O2—S1—N1—C1	−169.2 (3)	C5—C6—C7—C8	−170.6 (4)
O1—S1—N1—C1	55.7 (4)	C1—C6—C7—C8	9.6 (6)
C8—S1—N1—C1	−52.7 (3)	N2—C7—C8—S1	142.6 (3)
O2—S1—N1—C16	29.7 (5)	C6—C7—C8—S1	−39.0 (4)
O1—S1—N1—C16	−105.4 (4)	N2—C7—C8—Cl1	−95.6 (4)
C8—S1—N1—C16	146.1 (4)	C6—C7—C8—Cl1	82.8 (4)
C7—N2—N3—C9	178.1 (4)	O2—S1—C8—C7	170.5 (3)
C16—N1—C1—C6	−170.4 (4)	O1—S1—C8—C7	−61.3 (3)
S1—N1—C1—C6	28.6 (5)	N1—S1—C8—C7	56.1 (3)
C16—N1—C1—C2	8.4 (6)	O2—S1—C8—Cl1	48.0 (3)
S1—N1—C1—C2	−152.5 (3)	O1—S1—C8—Cl1	176.2 (2)
C6—C1—C2—C3	0.6 (7)	N1—S1—C8—Cl1	−66.4 (2)
N1—C1—C2—C3	−178.3 (4)	N2—N3—C9—C10	−179.2 (3)
C1—C2—C3—C4	−0.8 (8)	N3—C9—C10—C11	0.5 (6)
C2—C3—C4—C5	0.4 (8)	N3—C9—C10—C15	179.5 (4)
C3—C4—C5—C6	0.1 (7)	C15—C10—C11—O3	−178.6 (4)
C4—C5—C6—C1	−0.3 (6)	C9—C10—C11—O3	0.4 (6)
C4—C5—C6—C7	179.9 (4)	C15—C10—C11—C12	1.0 (6)
C2—C1—C6—C5	−0.1 (6)	C9—C10—C11—C12	−180.0 (4)
N1—C1—C6—C5	178.8 (4)	O3—C11—C12—C13	180.0 (4)
C2—C1—C6—C7	179.7 (4)	C10—C11—C12—C13	0.3 (7)
N1—C1—C6—C7	−1.4 (6)	C11—C12—C13—C14	−1.2 (8)
N3—N2—C7—C6	−177.7 (3)	C12—C13—C14—C15	0.7 (8)
N3—N2—C7—C8	0.7 (6)	C13—C14—C15—C10	0.8 (7)
C5—C6—C7—N2	7.9 (5)	C11—C10—C15—C14	−1.6 (6)
C1—C6—C7—N2	−171.9 (4)	C9—C10—C15—C14	179.3 (4)

### Hydrogen-bond geometry (Å, °)

**Table:**

*D*—H···*A*	*D*—H	H···*A*	*D*···*A*	*D*—H···*A*
O3—H3O···N3	0.82 (7)	1.98 (7)	2.682 (5)	143 (7)
C9—H9···O1^i^	0.95	2.55	3.394 (5)	148

Symmetry codes: (i) −*x*, −*y*+1, −*z*+1.
